# Hot Deformation Behavior and Processing Maps of SiC Nanoparticles and Second Phase Synergistically Reinforced Magnesium Matrix Composites

**DOI:** 10.3390/nano9010057

**Published:** 2019-01-03

**Authors:** Kaibo Nie, Zhihao Zhu, Kunkun Deng, Ting Wang, Jungang Han

**Affiliations:** 1 College of Materials Science and Engineering, Taiyuan University of Technology, Taiyuan 030024, China; zhihao.zhu@hotmail.com (Z.Z.); dengkunkun@tyut.edu.cn (K.D.); wangt_123@hotmail.com (T.W.); hanjgang@hotmail.com (J.H.); 2 Shanxi key Laboratory of Advanced Magnesium-Based Materials, Taiyuan University of Technology, Taiyuan 030024, China

**Keywords:** magnesium matrix nanocomposite, flow behavior, deformation mechanism, processing map

## Abstract

Magnesium matrix composites synergistically reinforced by SiC nanoparticles and second phases were prepared by 12 passes of multi-pass forging, varying the temperature. The effects of grain refinement and the precipitates on the hot deformation behavior were analyzed. Deformation zones which could be observed in the fine-grained nanocomposite before hot compression disappeared, and the trend of streamlined distribution for the precipitated phases was weakened. At the same compression rate, as the compression temperature increased, the number of precipitated phases decreased, and the grain size increased. For fine-grained nanocomposites, after the peak stress, there was no obvious dynamic softening stage on the stress–strain curve, and then the steady stage was quickly reached. The critical stress of the fine-grained nanocomposites was lower than that of the coarse-grained nanocomposites, which can be attributed to the large amounts of precipitates and significantly refined grains. The deformation mechanism of the coarse-grained nanocomposite was controlled by dislocation climb resulting from lattice diffusion, while the deformation mechanism for the fine-grained nanocomposite was dislocation climb resulting from grain boundary slip. The activation energy of the fine-grained nanocomposite was decreased, compared with the coarse-grained nanocomposite. The area of the workability region for the fine-grained nanocomposite was significantly larger than that of the coarse-grained nanocomposite, and there was no instability region at a low strain rate (0.001–0.01 s^−1^) under all deformation temperatures. The optimal workability region was 573 K /0.001–0.01 s^−1^ for the fine-grained nanocomposite, and the processing temperature was lower than the coarse-grained nanocomposite (623–673 K).

## 1. Introduction

Magnesium and magnesium-based alloys with high specific strength have attracted a wide range of research interests, due to their potential adaptation of energy and fuel savings in the transportation industry [1,2]. However, they also have several limitations such as poor corrosion resistance, creep strength, and workability, because of the hexagonal closed packed (HCP) structure and limited open slip systems, which may not meet the requirements for further application. To achieve the desired mechanical properties, the addition of different alloying elements to the magnesium is the most commonly used method [3]. Compared with conventional engineering materials such as aluminum alloy and steel, magnesium alloys are still inferior, despite their improved performance through alloying. In this case, nano-sized or micro-sized particle-reinforced magnesium matrix composites have been studied by more and more scholars. These materials have better creep resistance and friction resistance, as well as higher mechanical properties, both at ambient and elevated temperatures, compared with the matrix alloy [4,5,6]. A combination of ultrasonic vibration and semisolid stirring has been employed to fabricate SiC nanoparticle-reinforced AZ91-based composites in the author’s previous research [7]. Semisolid stirring can help to add the ceramic nanoparticles, while ultrasonic vibration can disperse nanoparticle agglomerations, leading to a uniform particle distribution.

Generally, the plastic forming ability at room temperature is limited for magnesium matrix composite due to the addition of brittle ceramic particles. Therefore, the magnesium matrix composites containing ceramic nanoparticles have to be deformed at elevated temperatures, in order to activate the additional slip system of the magnesium matrix [8]. The workability of the magnesium matrix nanocomposite can be described by using flow stress, which is related to the deformation extent, strain rate, and deformation temperature [9]. Studies have shown that the hot deformation behavior, metallurgical transformation kinetics, and processing maps are of greatest importance for the implementation of deformation [10,11,12]. Furthermore, processing maps are frequently employed by investigators to describe workability, while constructive equations are often used to understand the deformation mechanism. The hot deformation behavior, workability characteristics, and a processing map of a bimodal size SiCp/AZ91 magnesium matrix composite had been investigated by Zhou et al. [10], and the predicted characteristic microstructures by processing map were consistent with the experimentally observed microstructures. Srinivasan et al. [11] studied the processing maps of AZ31B magnesium alloy and its nanocomposite, and found that the optimum deformation conditions were the same for the AZ31B magnesium alloy and its nanocomposite. By processing maps, Prasad et al. [12] found that optimum processing conditions for Mg/nano-Al_2_O_3_ composite were at the deformation temperature range of 400–450 °C and at strain rates >0.1 s^−1^. According to the research results mentioned above, it is very meaningful to evaluate the hot deformation behavior, which is extremely important for optimizing the thermal deformation parameters. In our previous work [13], the hot deformation behavior and processing maps of coarse-grained nano-SiCp/AZ91 magnesium matrix composites prepared by the combination of semisolid stirring and ultrasonic vibration were compared to that of the AZ91 magnesium alloy. In addition, grain refinement has been proven to improve the deformation ability of magnesium alloys. Grain boundary slip can be prone to occur, to the coordinate deformation of fine-grained magnesium alloys during high-temperature deformation [14]. A literature search indicates that there are few studies on the high-temperature deformation behavior of fine-grained magnesium matrix composites reinforced by SiC nanoparticles and second phases. Therefore, it is of great significance to study the effect of SiC nanoparticles on the precipitation behavior of the second phase in the matrix alloy during high-temperature deformation, and to reveal the influence of SiC nanoparticles and second phases on the hot deformation behavior of fine-grained magnesium matrix nanocomposites.

In this paper, the as-cast coarse-grained nanocomposites were subjected to multi-pass forging to prepare fine-grained magnesium matrix nanocomposites. The influence of grain refinement and the precipitation of second phases on the deformed microstructure are analyzed. The effect of grain refinement and the precipitates on the deformation mechanism are investigated by calculating the activation energy and stress factor, according to the compressive stress–strain curves. Processing maps of the SiC nanoparticles and the second-phase synergistically reinforced magnesium matrix composites are constructed based on dynamic material modeling (DMM), to optimize the hot working domains. At the same time, the macroscopic morphology and the deformed structure of the sample after compression is used to determine the optimal deformation region.

## 2. Materials and Methods 

An AZ91 magnesium alloy with a composition of Mg-9.07Al-0.68Zn-0.21Mn was used as the matrix alloy, which was supplied by Northeast Light Alloy Company Limited (Harbin, China). SiC nanoparticles with an average particle size of 60 nm were selected as a reinforcement, which was supplied by Hefei Kaier Nanometer Energy & Technology Company Limited, China. The coarse-grained nano-SiCp/AZ91 composites (denoted as “CN”) were fabricated by the combination of ultrasonic vibration and semisolid stirring. The detailed preparation process had been described in detail in Ref. [7]. Before multi-pass forging, the coarse-grained composites were solution-treated at 420 °C for 24 hr to minimize the influence of the Mg_17_Al_12_ phase. The fine-grained nanocomposite (denoted as “FN”) was prepared by 12 passes of multi-pass forging, decreasing the temperature from 400 °C to 300 °C as described in detail in Ref. [15].

Cylindrical specimens 8 mm in diameter and 12 mm in height were machined from the fine-grained composite. A compression test was performed using a Gleeble 3500 thermomechanical simulator (Data Sciences International, New Brighton, Minnesota). In order to reduce the friction at the punch–specimen interface, the specimens were lubricated with graphite. All of the specimens were heated at a heating rate of 5 K/s by induction, and kept at a set temperature for 5 min, which with the aim of achieving a homogeneous temperature distribution. Once the strain reached the set strain value of 0.5, the compression process was terminated immediately, and all of the compressed specimens were water-quenched. The strain rate in the current compression experiments were set as 0.001, 0.01, 0.1, and 1 s^−1^, while the deformation temperature were selected as 523, 573, 623, and 673 K, which covers the hot deformation conditions for magnesium matrix composites containing nano-sized particles. 

The compressed specimens were cut in the center plane parallel to the compression direction, using wire electrical discharge machining. Microstructural analysis was carried out by using a MIRA 3XMU scanning electron microscope (SEM; Tescan, Brno, Czech Republic). The sectioned specimens to be analyzed were mounted, ground, polished, and etched, using acetic picral (5 mL acetic acid + 6 g picric acid + 10 mL H_2_O + 100 mL ethanol (95%)) prior to microstructure observation.

## 3. Results and Discussion

### 3.1. Microstructures

**Figure 1:**
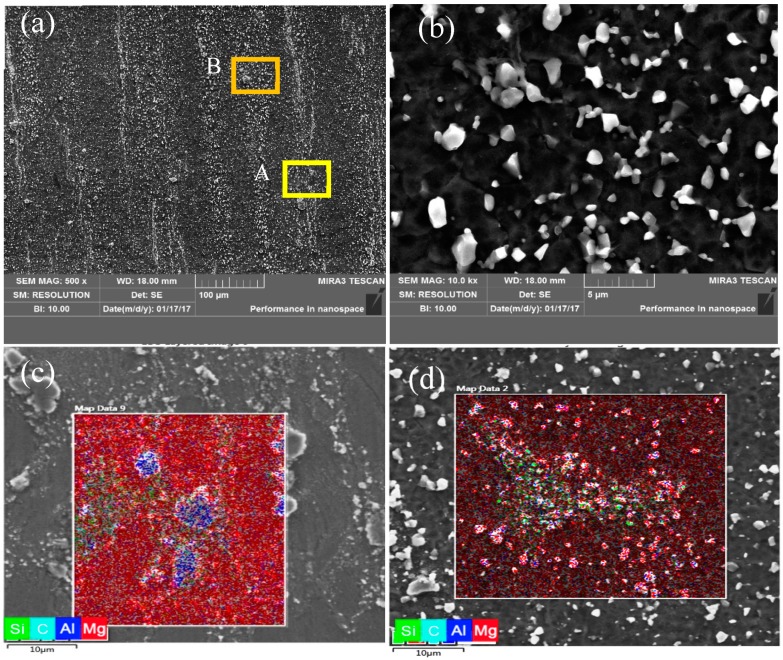
SEM images of the fine-grained nano-SiCp/AZ91 composite before hot compression: (**a**) low magnification, (**b**) high magnification, (**c**) EDS of area “A” in (**a**), (**d**) EDS of “B” in (**a**). (EDS indicates the energy dispersive spectroscopy.)

Figure 1 shows SEM images of the fine-grained nano-SiCp/AZ91 composite before hot compression. It can be found that after 12 passes of multi-pass forging with varying temperatures, the matrix grains are remarkably refined compared with the coarse-grained counterparts, as reported in our previous work [8]. The average grain size for the composite after 12 passes of multi-pass forging was 1.5 μm, and a large number of precipitated phases could be found in the matrix. During the multi-pass forging, the microstructure flows in different directions with the change of the applied load direction. The flow ability of the SiC nanoparticles is weaker than that of the matrix alloy during forging deformation, resulting in the appearance of a deformation zone. This indicates that a basal slip of the matrix alloy is more easily activated, and it can promote local deformation. As shown in Figure 1b, a mass of bulk and granular precipitated phases of Mg_17_Al_12_ were distributed at the grain boundaries. According to our previous research [15], with the decrease of deformation temperature from 673 K to 573 K, the average size of the precipitated phase distributed on the grain boundaries can reach the nanometer scale. These nano-sized precipitates can pin the grain boundaries and refine the grains. After 12 passes of multi-pass forging, the degree of dynamic recrystallization for the matrix was high, leading to further reduction of the grain size. Energy dispersive spectroscopy (EDS) of areas “A” and “B” further showed the distribution of the SiC nanoparticles and the precipitated second phases, as shown in Figure 1c,d. Although the SiC nanoparticle distribution in the composite was improved, there were still dense nanoparticle zones where coarse block-like precipitate phases also existed, as given in Figure 1c. Besides, there was a large amount of precipitated phases in the dense-nanoparticles zones, as shown in Figure 1d.

**Figure 2:**
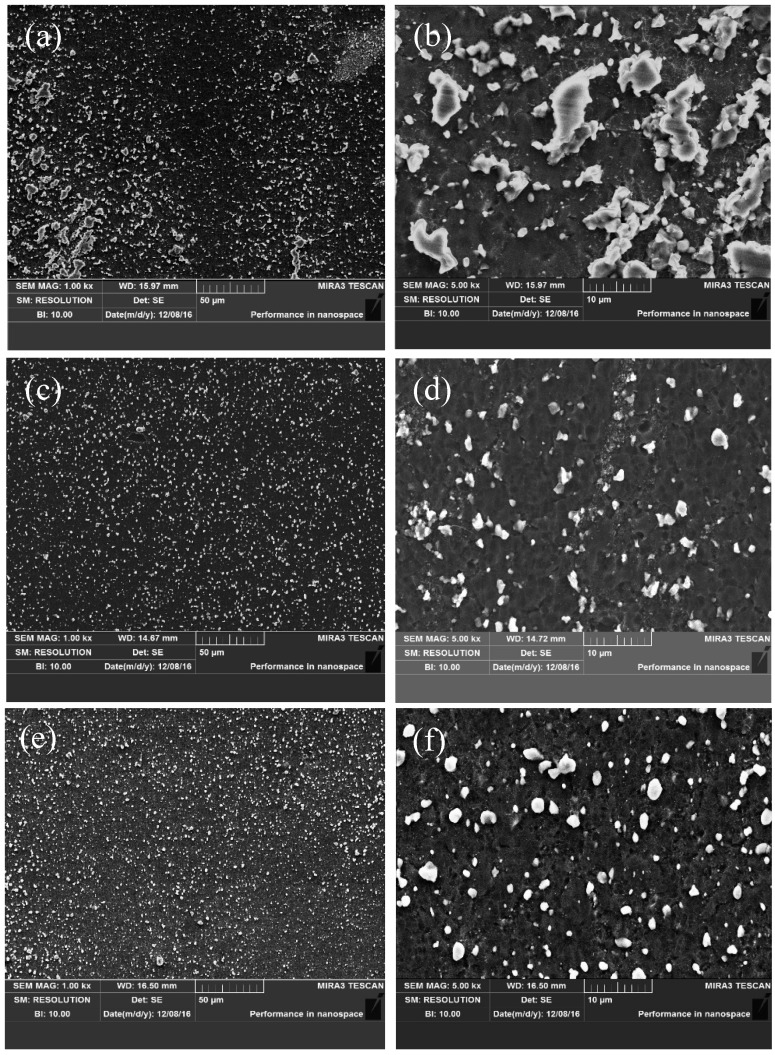
SEM images of fine-grained nanocomposites compressed at 573 K and different strain rates: (**a**,**b**) 0.001 s^−1^, (**c**,**d**) 0.01 s^−1^, (**e**,**f**) 1 s^−1^.

SEM images of fine-grained nanocomposites compressed at 573K and different strain rates are given in Figure 2. Before hot compression, the precipitated phase was streamlined parallel to the forging direction, while many deformation zones could be observed, as shown in Figure 1. With respect to the nano-SiCp/AZ91 composite after hot compression, as shown in Figure 2, the deformation zone disappeared, and the trend of streamlined distribution for the precipitated phases was weakened. At high strain rates, the precipitated phase was diffusely distributed. When the strain rate was 0.001 s^−1^, a dense region of the bulk precipitated phase, as well as granular precipitated phases, could be found in the matrix alloy. As the strain rates were 0.01 and 0.1 s^−1^, the shape of the precipitated phase did not change much, and all the second phases were granular. This was due to that the lower strain rate which provides a longer deformation time, leading to the segregation of precipitated atoms in solution, resulting in the growth of a large amount of precipitated phases in the original structure. At high strain rates, the nucleation rate of the precipitated phase was higher than the growth rate, leading to the refinement of precipitated phases.

**Figure 3:**
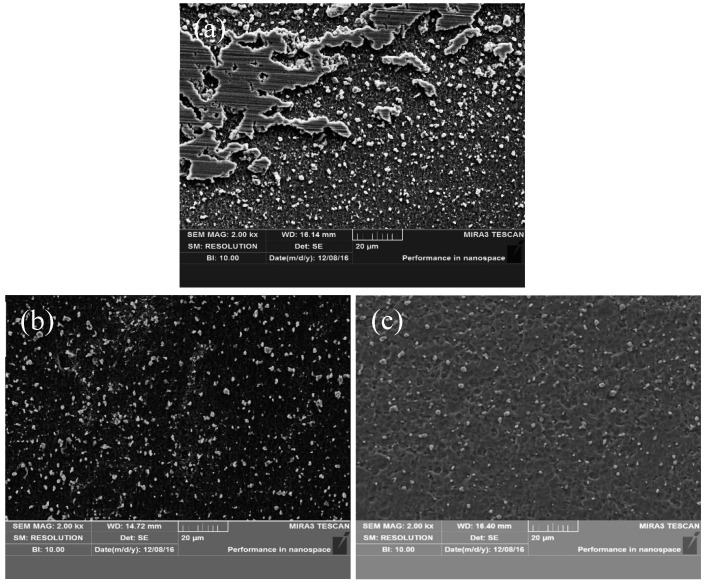
SEM images of fine-grained nanocomposites compressed at 0.01 s^−1^ and different temperatures: (**a**) 523 K, (**b**) 573 K, (**c**) 623 K.

Figure 3 shows SEM images of fine-grained nano-SiCp/AZ91 composites compressed at a strain rate of 0.01 s^−1^ and at different temperatures. It was found that at the same compression rate, as the compression temperature increased, the number of precipitated phases decreased and the grain size increased. Under the deformation condition of 523 K/0.01 s^−1^, the precipitated phase grew, and the number of the precipitates increased, due to the low deformation temperature and the long deformation time. When the compression temperature was increased from 573 to 623 K, the precipitated phases were re-dissolved into the matrix, due to high temperature, resulting in the disappearance of the bulk-like precipitated phase. At the same time, the nano-sized precipitates also dissolved back into the AZ91 matrix alloy. Most of the nano-sized precipitates were distributed at the grain boundaries; this can pin the grain boundary and hinder the grain growth. The re-dissolution of the precipitated phase reduces its pinning effect on the grain boundary, and weakens the hindrance of the grain growth, resulting in grain coarsening. Furthermore, as the compression temperature increases, both the atomic mobility and the migration rate of the grain boundary increase, leading to grain growth. Compared with the as-cast coarse-grained nanocomposite [8], there was no twinning, and a “streamline” structure composed of fine dynamic recrystallized grains was present in the fine-grained nanocomposite after hot compression, which indicates that grain refinement can significantly improve the deformability of the material. This is consistent with results regarding magnesium alloys, as reported by Mukai et al. [16]. Comparing the microstructures of the coarse-grained and fine-grained nanocomposites at the deformation condition of 573 K/0.01 s^−1^ [13], it can be found that the number of particles in the fine-grained nanocomposite was significantly larger than that of the coarse-grained nanocomposite. There was a large number of SiC nanoparticles distributed on the grain boundaries of the fine grains in the fine-grained nanocomposite. Since the nanoparticles had strong pinning effects on the grain boundaries, the grains of the fine-grained nanocomposites were significantly smaller than those of the coarse-grained nanocomposites after hot compression.

### 3.2. Compressive True Stress–True Strain Curves

**Figure 4:**
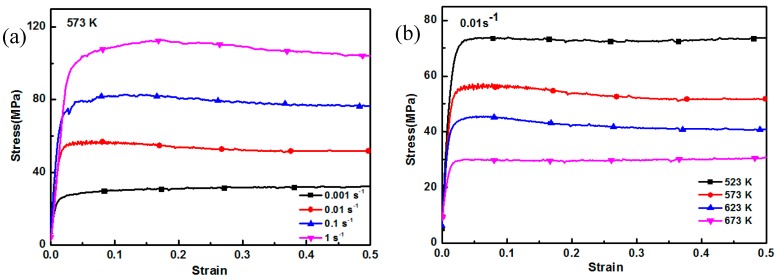
Compressive true stress–strain curves of the fine-grained nanocomposite: (**a**) 573 K, (**b**) 0.01 s^−1^.

**Figure 5:**
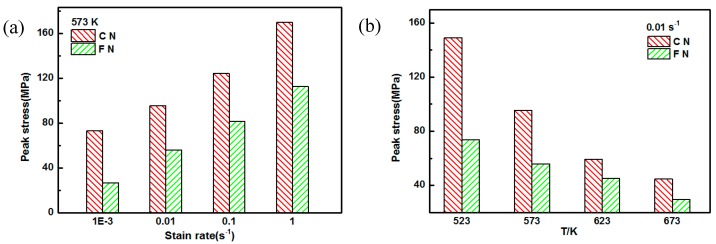
The peak stress of the coarse-grained nanocomposite (CN) and the fine-grained nanocomposite (FN): (**a**) 573 K, (**b**) 0. 01 s^−1^.

Figure 4 shows the compressive true stress–true strain curves of fine-grained nanocomposites under different compression conditions. As the strain rate decreased or the deformation temperature increases, the flow stress for the fine-grained nanocomposites gradually decreased. In our previous work, the stress–strain curves of both the AZ91 alloy and the coarse-grained nanocomposites were typical compression stress–strain curves, and they consisted of four stages: work hardening stage, transition stage, softening stage, and steady stage [8,13]. For fine-grained nanocomposites, after the peak stress, there was no obvious dynamic softening stage on the stress–strain curve, and then the steady stage was quickly reached. This indicates that a balance between work hardening and dynamic softening is easily achieved. Compared with the stress–strain curves of coarse-grained nanocomposites [8], the flow stress of the fine-grained nanocomposites was lower under different compression conditions. This is because the grain size of fine-grained nanocomposites was only 1.5 μm and the microstructure distribution was uniform after 12 passes of multi-pass forging. In contrast, the grain size of the coarse-grained nanocomposites was 96 μm [13]. At high temperature compression, the deformation degree for each grain in the alloy containing fine grains has no obvious difference, resulting in uniform deformation. Coordinated deformation of the grains can occur under lower compressive stress, improving the deformability. Figure 5 shows the peak stress of the coarse-grained nanocomposite and the fine-grained nanocomposite under different compression conditions. It was found that as the deformation temperature increased and the strain rate decreased, the peak stress of the fine-grained nanocomposite decreased, which was lower than that of the coarse-grained nanocomposite. This is mainly because the softening effect caused by the fine grains is stronger than the dispersion strengthening effect and the dislocation strengthening effect of the SiC nanoparticles, resulting in the lower peak stress of the fine-grained nanocomposite, compared with that of the coarse-grained nanocomposite. As shown in Figure 5b, the peak stresses of the coarse-grained nanocomposite were significantly higher than the fine-grained nanocomposite, under the deformation condition of 523 K/0.01 s^−1^. This can be attributed to the appearance of large amounts of twins in the coarse-grained nanocomposite, which were not found in the fine-grained nanocomposite.

**Figure 6:**
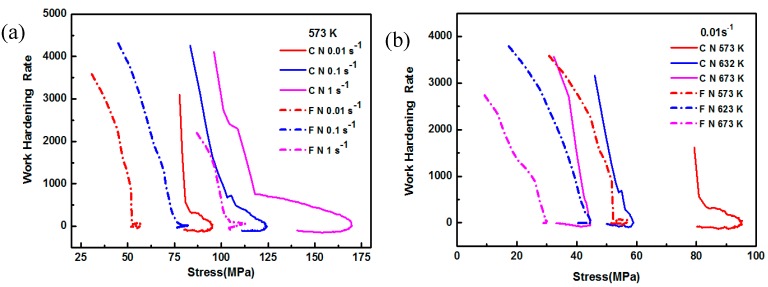
Schematic charts of θ – σ for the coarse-grained nanocomposite (CN) and the fine-grained nanocomposite (FN): (**a**) 573 K, (**b**) 0. 01 s^−1^.

**Figure 7:**
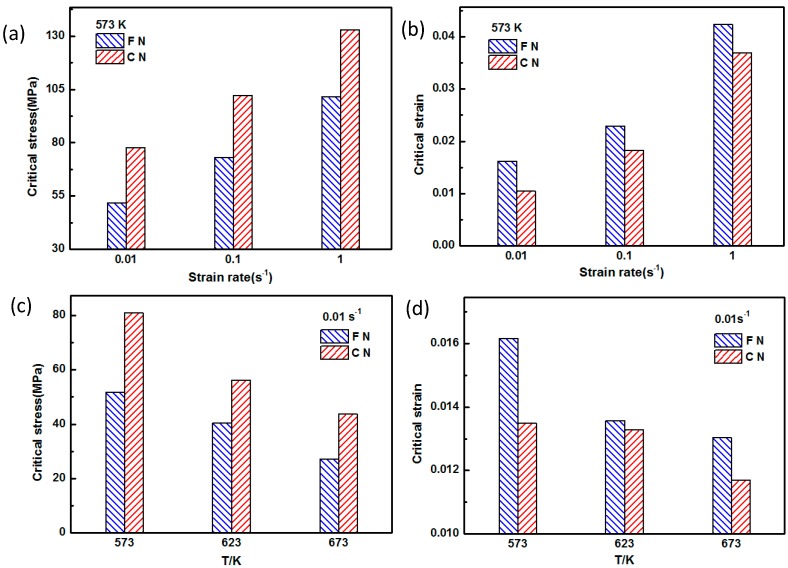
Critical stress and strain of the coarse-grained nanocomposite (CN) and the fine-grained nanocomposite (FN): (**a**,**b**) 573 K, (**c**,**d**) 0.01 s^−1^.

Figure 6 shows the relationship between θ and σ of the coarse-grained nanocomposite and the fine-grained nanocomposite. This further demonstrates that the peak stress of the fine-grained nanocomposites is lower than that of the coarse-grained nanocomposite. Poliak et al. [17] has described the (d*θ*/d*σ*)/*σ* curve based on the *θ*–*σ* curve, and the stress at the lowest point on the curve is the critical stress. Then, the critical strain is determined on the stress–strain curve according to the critical stress. In order to study the effects of grain size and precipitates on the critical conditions of dynamic recrystallization, the critical strains of the coarse-grained nanocomposite and fine-grained nanocomposite during thermal compression were compared, as shown in Figure 7a,c. The critical strain can be determined according to the critical stress, as shown in Figure 7b,d. It was seen that the critical strains of both the fine-grained nanocomposites and the coarse-grained nanocomposites decreased with the increase of deformation temperature, and the decrease of strain rate. This is because at high temperatures and low strain rates, the mobility of dislocations and the migration ability of the grain boundaries are strong, making dynamic recrystallization more likely to occur at lower temperatures. As shown in Figure 7a,c, the critical stress of the fine-grained nanocomposites was lower than that of the coarse-grained nanocomposites. This phenomenon was caused by two reasons. First, the fine-grained nanocomposite before hot compression contained a large amount of precipitated phase Mg_17_Al_12_. Some of the nano-sized precipitates could hinder the movement of dislocations during hot compression, resulting in a strengthening effect. However, the Mg_17_Al_12_ phase is not a stable hard particle, and it can be dissolved and softened at high temperature; its effect on increasing the deformation resistance was not obvious. Second, the grain size of the coarse-grained nanocomposite was significantly larger than the fine-grained nanocomposite before hot compression, and the grain refinement had a strong weakening effect on deformation resistance. The combined effects of the above facts led to the decrease in the critical stress of the fine-grained nanocomposite. The critical strains of the fine-grained nanocomposite were higher than the coarse-grained nanocomposites, with varying deformation temperatures or strain rates, as shown in Figure 7b,d. Li et al. [18] compared the AZ91 alloy with fine-grained double-size magnesium matrix composites under hot compression conditions, and found that grain refinement can promote the formation of dynamic recrystallization. 

In our previous study [13], due to the addition of SiC nanoparticles, the grain size of coarse-grained nanocomposites is smaller, while the critical strain of dynamic recrystallization is lower than that of the AZ91 alloy. However, the critical strain of the fine-grained nanocomposite in the current study was higher than that of the coarse-grained nanocomposite, indicating that this phenomenon is caused by the precipitated phase. The presence of small-sized particles or second phases with a small particle spacing and uniform distribution in the magnesium matrix composite will hinder or delay the progress of dynamic recrystallization for the magnesium matrix. Humphreys et al. [19] have shown that small-sized particles can pin small-angle and high-angle grain boundaries, hindering the migration of grain boundaries. Before the hot compression, there are many nano-sized precipitated phases on the grain boundaries for the fine-grained nanocomposite. During the hot compression, the deformation near these precipitates phases is not uniform, leading to the generation of a large number of dislocations. The dislocation motion can be hindered by the nanoparticles distributed on the grain boundaries, resulting in the formation of dislocation entanglement, which provides favorable conditions for the nucleation of dynamic recrystallization. At the same time, it can hinder the grain boundary bowing, and it has a strong pinning effect on the grain boundary, hindering the progress of dynamic recrystallization. As a result, the critical strain of the fine-grained nanocomposite was higher than that of the coarse-grained nanocomposite.

### 3.3. Constitutive Analysis

Constitutive equations can usually be used to describe the relationship between strain rate and flow stress during thermal deformation [20]. The three types of constitutive equations include power, exponential, and hyperbolic constitutive equations, which can be described by Equations (1)–(3) [21]:

(1) $$\dot{\varepsilon }=A_{1}\sigma^{n_{1}}\exp\left(-\frac{Q}{RT}\right)$$

where *A*_1_ stands for the material constant, $\dot{\varepsilon }$ stands for the strain rate, *σ* represents flow stress, *n*_1_ are the stress exponents, *Q* stands for the activation energy during hot deformation, and *R* and *T* represent the gas constant (8.31 J·mol^−1^·K^−1^) and absolute temperature, respectively.

(2) $$\dot{\varepsilon }=A_{2}\exp\left(\beta \sigma \right)\exp\left(-\frac{Q}{RT}\right)$$

where *A*_2_ and *β* are material constants, and other parameters have the same meaning as Equation (1).

(3) $$\dot{\varepsilon }=A_{3}{\left(\sinh(\alpha \sigma )\right)}^{n}\exp\left(-\frac{Q}{RT}\right)$$

where *A*_3_ and *α* are material constants, *n* stands for the stress exponents, and other parameters have the same meanings as in Equation (1). 

The Zener–Hollomon parameter Z, which combines the influence of the strain rate $\dot{\varepsilon }$ and the temperature *T*, is described by Equation (4):

(4) $$Z=\dot{\varepsilon }\exp(\frac{Q}{RT})$$

Normally, the power law is applied under low-stress conditions, the exponential law is suitable for use under high-stress conditions, and the hyperbolic sine law can be used over a wider stress range. Therefore, the hyperbolic sinus law was selected to evaluate the hot deformation behavior of the fine-grained nano-SiCp/AZ91 composite, and the stress value was determined, with a strain of 0.5. The high-temperature deformation mechanism of the current fine-grained nanocomposite can be determined by calculating the values of the activation energy *Q* and the stress exponent *n*. The calculation steps are as follows:

Equation (5) can be obtained by taking the natural logarithm on both sides of Equation (1):

(5) $$\ln\dot{\varepsilon }=n_{1}\ln\sigma +\ln A_{1}-\frac{Q}{RT}$$

**Figure 8:**
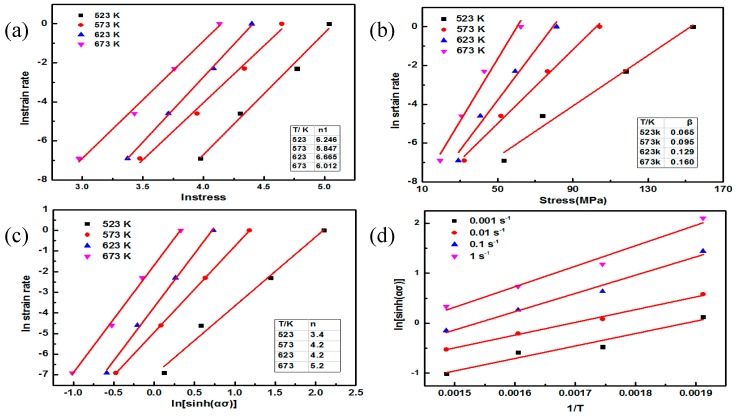
Linear fitting of the fine-grained nanocomposite: (**a**) ln$\dot{\varepsilon }$−lnσ, (**b**) ln$\dot{\varepsilon }$-σ, (**c**) $\dot{\varepsilon }$-ln (sinh (ασ)), (**d**) ln (sinh (ασ)) − 1/T.

Then the value of the stress exponent *n*_1_ is obtained by calculating the plot of the curve ln$\dot{\varepsilon }$ −lnσ, as shown in Figure 8a. 

Equations (2) and (3) are processed in the same way as the Equation (1), as expressed by Equations (6) and (7):

(6) $$\ln\dot{\varepsilon }=\sigma \beta +\ln A_{2}-\frac{Q}{RT}$$

(7) $$\ln\dot{\varepsilon }=n\ln\left(\sinh(\alpha \sigma )\right)+\ln A_{3}-\frac{Q}{RT}$$

By fitting the curve of ln$\dot{\varepsilon }$ −σ according to Equation (6), the value of the material constant *β* is calculated, as shown in Figure 8b. Based on the formula *α* = *n*_1_/*β*, the value of α can be determined. The value of α was then substituted into Equation (7), and the value of the stress exponent *n* can be determined by fitting the curve of ln$\dot{\varepsilon }$ −ln(sinh(ασ)), as given in Figure 8c. After the deformation of Equation (7), the following Equation (8) can be obtained:

(8) $$\ln\left(\sinh(\alpha \sigma )\right)=\frac{Q}{nRT}+\frac{\ln\dot{\varepsilon }}{n}-\frac{\ln A_{3}}{n}$$

Figure 8d demonstrates the linear regression analysis performed on the curve of ln(sinh(ασ))−1/T. Table 1 gives the values of stress exponent *n* for the fine-grained nanocomposite. It can be seen from Table 1 that stress exponent *n* increases as the deformation temperature increases, for the fine-grained nanocomposite. A previous study has shown that the variation of the stress exponent *n* represents different deformation mechanisms [22]: when the value of *n* is 2, 3, 5, or 8, they represent the grain boundary slide, dislocation glide, dislocation climb, and the constant substructure model, respectively. Thus, the value of the stress exponent *n* is 3.4 at a deformation temperature of 523 K, and the deformation mechanism can be determined as the dislocation glide mechanism. In contrast, the value of the stress exponent *n* is close to 5 as the deformation temperature ranges from 573 to 673 K, Thus, the deformation mechanism under this situation is the dislocation climbing mechanism, which is similar to the coarse-grained nanocomposite. Based on the Equation (3), the activation energy *Q* can be described by Equation (9):

(9) $$Q=R(\frac{\partial \ln\left(\sinh(\alpha \sigma )\right)}{\partial \left(1/T\right)})\dot{\varepsilon }\;(\frac{\partial \ln\overset{\cdot }{\varepsilon }}{\partial \ln\left(\sinh(\alpha \sigma )\right)}{)}_{T}$$

As shown in Table 2, the activation energy *Q* can be obtained by the linear regression analysis of Equation (9) (Figure 8c,d). In Table 2, it is found that at the same strain rate, when the fine-grained nanocomposite was compressed at a high temperature interval of 623–673 K, the value of activation energy *Q* of the fine-grained nanocomposite was significantly lower than that at low-temperature compression. At the same deformation temperature, the value of the activation energy *Q* decreases as the strain rate decreases. When the strain rate varies from 0.01 to 0.001 s^−1^, the value of the activation energy *Q* for the fine-grained nanocomposite ranges from 70 to 110 kJ/mol, which is close to the activation energy of grain boundary diffusion for pure magnesium (Q_gb_ = 82–105 kJ/mol) [23]. When the strain rate varies from 0.1 to 1 s^−1^ at a temperature interval of 523–623K, the value of the activation energy *Q* for the fine-grained nanocomposite is close to the activation energy of lattice diffusion for pure magnesium (*Q_L_* = 135 kJ/mol) [23]. Besides, when the strain rate varies from 0.1 to 1 s^−1^ at a deformation temperature of 673 K, the value of activation energy *Q* for the fine-grained nanocomposite is close to the activation energy of grain boundary diffusion for pure magnesium (*Q_gb_* = 82–105 kJ/mol). The value of the stress exponent *n* at 523 K is 3 for the fine-grained nanocomposite, so the deformation mechanism is controlled by dislocation glide, resulting from grain boundary diffusion under the processing conditions of 523 K/0.001–0.01 s^−1^, and dislocation glide resulting from lattice diffusion under the processing conditions of 523 K/0.01–1 s^−1^. When the deformation temperature ranges from 573 to 623 K, the value of *n* is close to 5. The deformation mechanism of the fine-grained nanocomposite can be determined as dislocation climb resulting from grain boundary diffusion under the processing conditions of 573–623 K/0.001–0.01 s^−1^ and dislocation climb resulting from lattice diffusion under the processing conditions of 573–623 K/0.1–1 s^−1^. In contrast, the value of *n* is close to 5 at a deformation temperature of 673 K, which indicates that the deformation mechanism is controlled by dislocation climb resulting from grain boundary slip. 

**Figure 9:**
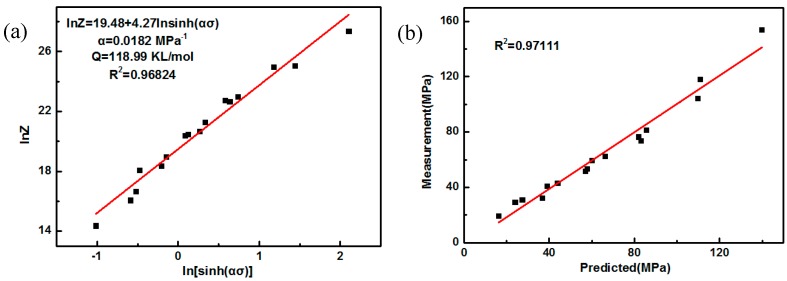
Linear fitting of (**a**) lnZ-ln(sinh (ασ)), (**b**) theoretical–actual stress for the fine-grained nanocomposite.

As shown in Figure 9a, the value of *A* and the constitutive equation of parameters can be evaluated using linear fitting curves of lnZ-𝑙𝑛(𝑠𝑖𝑛ℎ (𝛼𝜎)), according to Equations (3) and (4). Substituting the deformation temperature and strain rate into the constitutive equation, a theoretical stress value corresponding to the deformation condition can be obtained. The theoretical value and the actual value of the flow stress are illustrated and fitted, as shown in Figure 9b. The linearly dependent coefficient *R* between the measured value and the calculated value is higher than 0.95, and close to 1, as can be seen in Figure 9. This high-fitting degree indicates that the hyperbolic sine can accurately describe the deformation mechanism of the fine-grained nanocomposite.

In order to characterize the effects of grain size and the precipitated phase on the deformation mechanism of the fine-grained nanocomposite at high temperatures, the stress exponent *n* and the deformation activation energy *Q* of the coarse-grained nanocomposite and the fine-grained nanocomposite was compared, as shown in Table 3. It can be seen from Table 3 that the deformation mechanism of the coarse-grained nanocomposite was controlled by dislocation climb resulting from lattice diffusion. With regard to the fine-grained nanocomposite, the deformation mechanism was dislocation climb resulting from grain boundary slip, and the activation energy was decreased compared with the coarse-grained nanocomposite. This phenomenon can be attributed to the grain refinement. The fine-grained nanocomposite with average grain size of 1.5 μm contained a large number of grain boundaries, and so grain boundary slip and coordinate deformation are prone to occur. Thus, for the fine-grained nanocomposite, the decrease in the activation energy due to grain refinement far exceeds the increase resulting from the hindering of dislocation motion by the precipitated phase.

### 3.4. Processing Maps

The relationship between the flow stress *σ* and the strain rate $\dot{\varepsilon }$ during the hot deformation can be described by Equation (10) [24]:

(10) $$\sigma =K{\dot{\varepsilon }}^{m}$$

The cubic spline function was then used to fit the curve of lnσ−ln$\dot{\varepsilon }$. The accuracy of the corresponding value m can be guaranteed by the coefficient. In this case, under the same temperature, the power dissipation factor can be described by Equation (11) [25]:

(11) $$m=\ln\sigma /\ln\dot{\varepsilon }$$

During the DMM, the dissipative efficiency of power can be used to evaluate the property for power dissipation resulting from microstructure change [25], which can be represented by Equation (12):

(12) $$\eta =\frac{2m}{2m+1}$$

where the value of *m* is variable, while η stands for the proportional relation between the energy consumed by the microstructures’ evolution, and the linear dissipative energy during deformation. The power dissipation map in the two-dimensional plane is generally described as an iso-efficiency counter map, and it can be obtained based on the deformation temperature and strain rate. The onset of flow instability can be defined by the following criterion, as given by Equation (13) [25]:

(13) $$\zeta \left(\dot{\varepsilon }\right)=\frac{\partial \ln\left(\frac{m}{m+1}\right)}{\partial \ln\dot{\varepsilon }}+m\leq 0$$

where $\zeta \left(\dot{\varepsilon }\right)$ represents the instability parameter determined by the strain rate and deformation temperature. A positive value of $\zeta \left(\dot{\varepsilon }\right)$ indicates the appearance of a steady state during the deformation processing. On the contrary, a negative value of $\zeta \left(\dot{\varepsilon }\right)$ confirms the occurrence of flow instability. 

**Figure 10:**
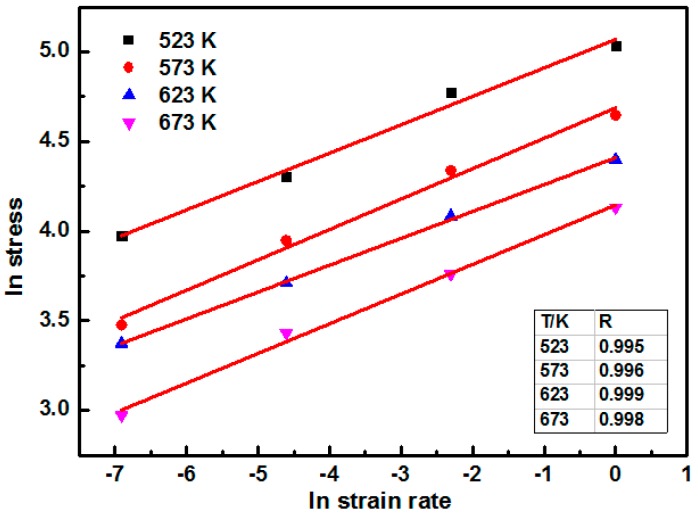
Linear fitting of ln$\dot{\varepsilon }$-lnσ for the fine-grained nanocomposite.

According to the theory for the processing map, before drawing the processing map of the fine-grained nano-SiCp/AZ91 composite, it is necessary to fit the straight line using the strain rate and the flow stress. The value of the fitting coefficient can be used to judge the reliability of the selected values. Figure 10 shows the linear fitting of ln$\dot{\varepsilon }$-lnσ for the fine-grained nanocomposite. Under different compression conditions, the values of fitting coefficient *R* for the strain rate and stress were larger than 0.99. This high fitting degree indicates that the DMM can be used to draw the processing map of the fine-grained nanocomposite.

**Figure 11:**
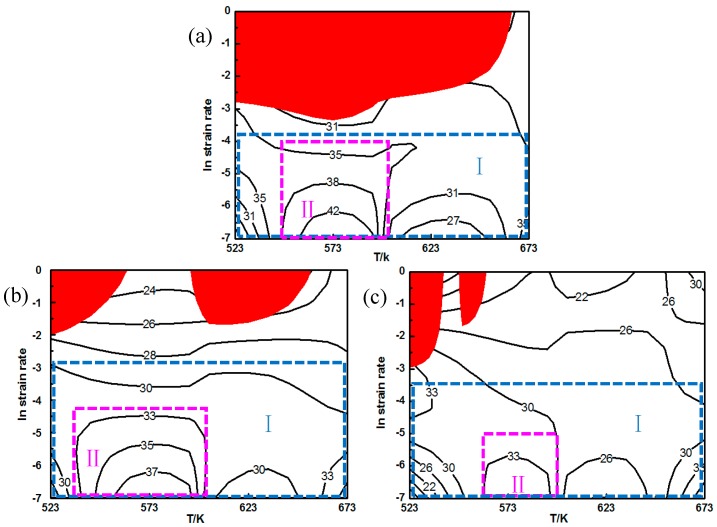
Processing maps of the fine-grained nanocomposite compressed at different strains: (**a**) 0.3, (**b**) 0.4, (**c**) 0.5.

Figure 11 shows the processing maps of the fine-grained nanocomposite at different strains. The red area in Figure 11 stands for the instability area, and the number on the equivalent line represents the value of the power dissipation efficiency (%). It can be found that the area of the instability zone is significantly reduced with the increase of the strain, and the distribution of the instability zone changes significantly from a concentrated distribution to a dispersed distribution. This indicates that the fine-grained nanocomposites are sensitive to the corresponding strain. When the strain is 0.2, a large area of instability occurs in the processing map. The instability region appears at a high strain rate of 0.1–1 s^−1^ under different deformation temperatures, as shown in Figure 11a. When the strain is increased to 0.3, as shown in Figure 11b, the areas of instability for the fine-grained nanocomposite is significantly reduced, which is mainly distributed at a high strain rate under deformation temperatures of 523 K and 623 K. When the strain reaches a maximum test strain of 0.5, the area of the instability zone is further reduced. The instability zone is only distributed at a high strain rate under a temperature of 523K, as shown in Figure 11c. In Figure 11, there is a large range of workability region I (the area contained in the blue rectangular frame in the figure) in the processing maps of the fine-grained nanocomposite at different strains. This indicates the fine-grained nanocomposite possesses a large processing temperature range, and can be processed in a temperature range of 523–673 K at low strain rates. In region I, the value of the power dissipation efficiency decreases with the decrease of the strain rate. In contrast, when the deformation temperature ranges from 573 to 673 K, the value of power dissipation efficiency increases with a decreasing strain rate. This indicates that the power dissipation efficiency of the fine-grained nanocomposite is sensitive to the deformation temperature. It can be seen from Figure 11 that in region II (the area enclosed by the red dotted rectangular wireframe), the value of the power dissipation efficiency is in the range of 33–42%, indicating that dynamic recrystallization (DRX) occurs in the fine-grained nanocomposite. In Figure 11, all of the peak values of power dissipation efficiency appear in region II at a corresponding deformation temperature of 573 K. The peak values of the power dissipation efficiency at strains of 0.2, 0.3, and 0.5 are 42%, 35%, and 33%, respectively. It can be seen that the peak values of the power dissipation efficiency of the fine-grained nanocomposite decrease as the strain increases. There is no rheological instability, and the power dissipation efficiency is high within region II, and so this area can be regarded as the best workability region for the fine-grained nanocomposite.

**Figure 12:**
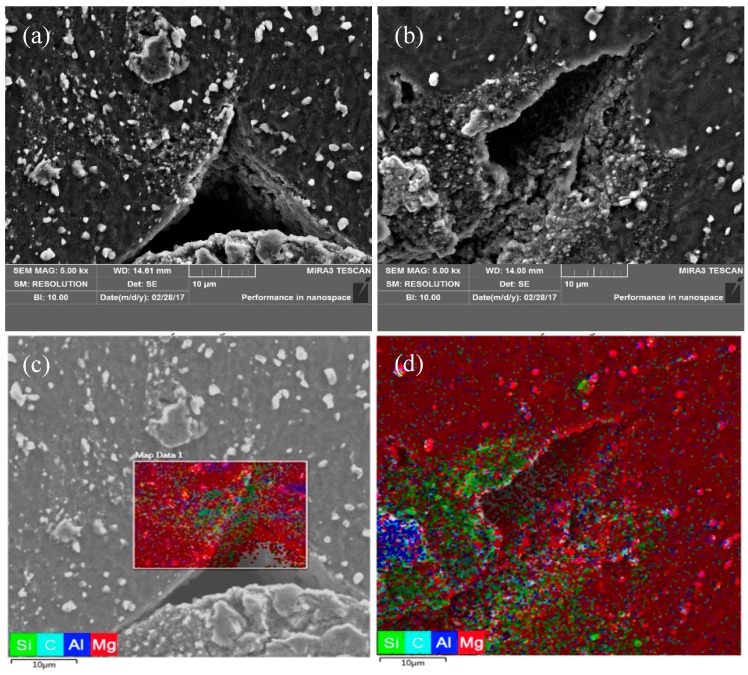
SEM of the fine-grained nanocomposite compressed at (**a**) 523 K and 1 s^−1^, (**b**) 573 K and 1 s^−1^, (**c**,**d**) the EDS of (**a**,**b**), respectively.

**Figure 13:**
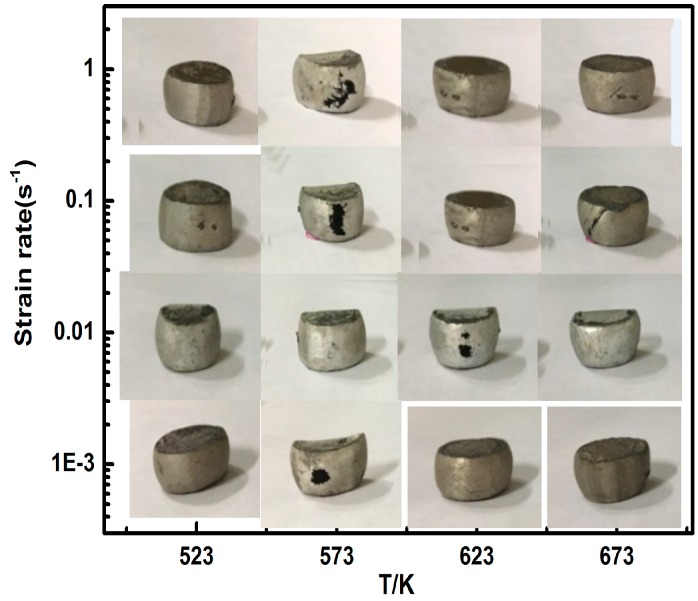
External crack morphologies of fine-grained nanocomposites after hot compression.

Figure 12 shows SEM images of crack morphology for the fine-grained nanocomposite compressed at 523 K/1 s^−1^ and 573 K/1 s^−1^. EDS analysis of the cracked region showed that there was a large number of precipitated phases around the crack. Besides, the cracked region occurred in the dense-nanoparticles zone, which indicates that the dense-nanoparticles zone is the main region of crack initiation. Figure 13 shows the macroscopic morphology of the fine-grained nanocomposite under different compression conditions. It was found that there was no macroscopic cracking on the surface of the fine-grained nanocomposites in all of the regions at low strain rates (0.01–0.001 s^−1^). Macroscopic cracking only occurred under the processing condition of 673 K/0.1–1 s^−1^, which was consistent with the processing map of the fine-grained nanocomposite.

**Figure 14:**
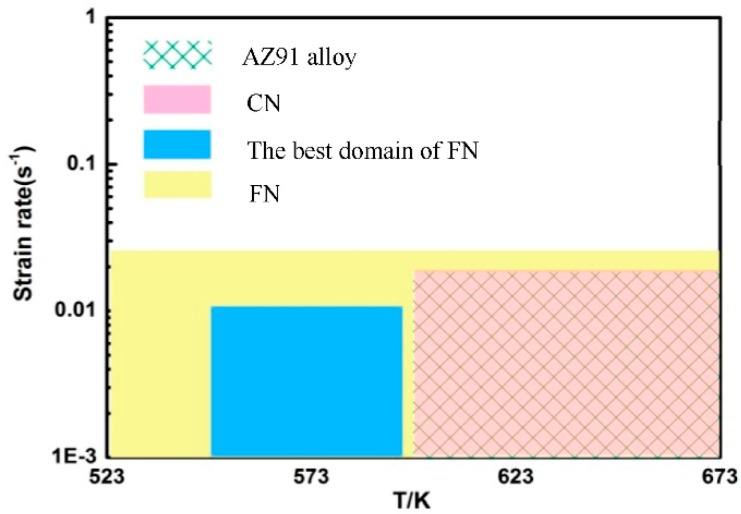
The safe area of the AZ91 alloy, the as-cast coarse-grained nanocomposite (CN), and the fine-grained nanocomposite (FN) at a strain of 0.5.

In order to reveal the effects of grain size and precipitates on the distribution of the processing map, the workability region in the processing map for the fine-grained nanocomposite was compared with the as-cast coarse-grained nanocomposite and other published literatures. The processing map for the coarse-grained nanocomposite has been reported in our previous work [13]. When the strain is 0.2, the instability areas of both the coarse-grained nanocomposite and the fine-grained nanocomposite are distributed at the high strain rate zones. The instability can be found at almost all deformation temperatures for the fine-grained nanocomposite, while only at 523 K and 623 K for the coarse-grained nanocomposite. When the strain is increased to 0.3, the instability area of the fine-grained nanocomposite is significantly smaller than that of the coarse-grained nanocomposite. The instability can be found at almost all strain rates under low deformation temperatures for the coarse-grained nanocomposite, and at only 523 K and 623 K at high strain rates for the fine-grained nanocomposite. As the strain further increases to 0.5, the instability area for the fine-grained nanocomposite is very small, which is only within the partial instability region of 523–573 K/0.1–1 s^−1^ for the coarse-grained nanocomposite. Based on the above analysis, the fine-grained nanocomposite and the coarse-grained nanocomposite have the same instability region. Figure 14 shows the safe areas of the AZ91 alloy, the as-cast coarse-grained nanocomposite, and the fine-grained nanocomposite, at a strain of 0.5. As can be seen from Figure 14, the optimum workability region for the AZ91 alloy is the same as the coarse-grained nanocomposite. The area of the workability region for the fine-grained nanocomposite is significantly larger than that of the coarse-grained nanocomposite. There is no instability region at a low strain rate (0.001–0.01 s^−1^) under all deformation temperatures. In contrast, the processing area is 623–673 K /0.001–0.01 s^−1^ for the coarse-grained nanocomposite. Moreover, the optimal workability region is 573 K /0.001–0.01 s^−1^ for the fine-grained nanocomposite, which is lower than the processing temperature for the coarse-grained nanocomposite (623 K–673 K). This is because the degree of DRX is high in the workability region of the coarse-grained nanocomposite. For example, the grain size distribution of the coarse-grained nanocomposite is relatively uniform at processing conditions of 623 K/0.01 s^−1^, the grain boundary is relatively flat, and the structure is relatively uniform. With regard to the fine-grained nanocomposite, the DRX degree is high at an optimum temperature of 573 K. At this deformation temperature, a large number of nano-sized precipitates are distributed at the grain boundaries, and the degree of grain coarsening is small. At 673 K, the degree of DRX of the fine-grained nanocomposite is high, and the degree of remelting of the nano-sized precipitates is also large. As a result, the degree of the hindrance of grain boundary migration is drastically weakened, and grain coarsening is severe.

**Figure 15:**
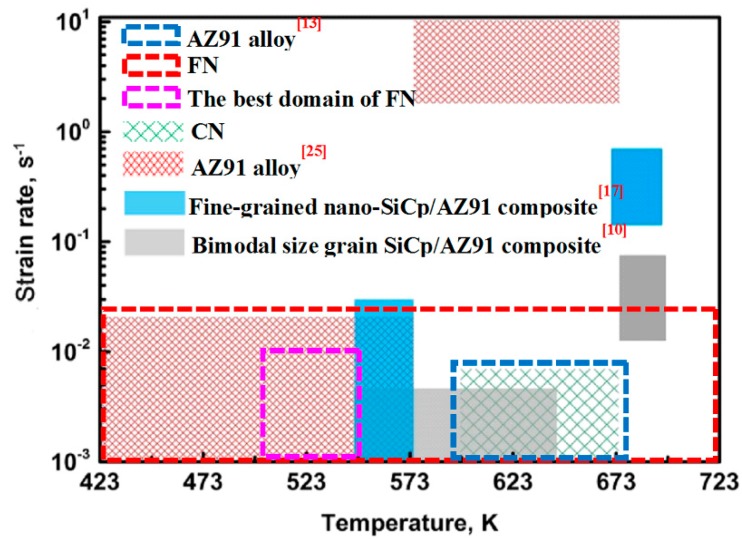
Safe area of different materials at a strain of 0.5.

Figure 15 shows the safe area of different materials at strain of 0.5. It can be found that the workability region for the AZ91 alloy powder compacts is different from that of the commercial AZ91 alloy fabricated by die casting [26], which proves that forming conditions can affect the distribution of the processing map. Compared with the bimodal sized SiCp/AZ91 composite [10], the deformation temperature corresponding to the workability region of the coarse-grained nanocomposite is higher, which may be caused by the difference in grain size. Before hot compression, the initial grain size is only 8 μm for the bimodal-sized composite, while the grain size of the coarse-grained composite is 95 μm, which is significantly higher than the bimodal-sized composites. The workability region between the bimodal-sized composite and the fine-grained nano-SiCp/AZ91 composite was compared [17]. It was found that the workability region of the fine-grained nano-SiCp/AZ91 composite was larger, and that the deformation temperature corresponding to the optimal workability region waslower than that of the bimodal-sized composite. Since the difference in grain size between the fine-grained nano-SiCp/AZ91 composite and the bimodal-sized composite is small, the phenomenon may be caused by reinforcement particles/precipitates. The nano-sized precipitated phase, which can pin the grain boundaries of fine-grained nano-SiCp/AZ91 composite, dissolves at high temperature, and it only works under a low deformation temperature. However, the reinforcement of the bimodal-sized composite is submicron SiC particles, which is a high-temperature stable phase, and it can hinder the grain boundary migration at high temperatures and refine the DRX grains.

## 4. Conclusions

In the present work, hot deformation behavior and processing maps of the SiC nanoparticles and second-phase synergistically reinforced magnesium matrix composite are created. The following conclusions are drawn:

(1) As the compression temperature increases at the same compression rate, the number of precipitated phase decreases, while the grain size increases for the fine-grained nano-SiCp/AZ91 composite. The deformation zones in the fine-grained nanocomposite disappears after hot compression, and the trend of streamlined distribution for the precipitated phases is weakened

(2) After the peak stress, there is no obvious dynamic softening stage on the stress–strain curve for the fine-grained nano-SiCp/AZ91 composite. The critical stress of the fine-grained nanocomposites is lower than that of the coarse-grained nanocomposites, which can be attributed to the large amounts of precipitates and significantly refined grains.

(3) A hyperbolic sine can accurately describe the deformation mechanism of the fine-grained nano-SiCp/AZ91 composite from the present work. The dominant deformation mechanism is dislocation climb, resulting from grain boundary slip for the fine-grained nanocomposite. 

(4) There is no instability region at a low strain rate (0.001–0.01 s^−1^) under all deformation temperatures for the fine-grained nano-SiCp/AZ91 composite, and its optimal workability region is 573 K/0.001–0.01 s^−1^. The area of the workability region for the fine-grained nanocomposite is significantly larger, while the processing temperature is lower than for the coarse-grained nanocomposite.

## Figures and Tables

**Table 1:** The *n* values for the fine-grained nanocomposite.

T/K	523	573	623	673
CN [13]	3.1	4.8	5.7	5.8
FN	3.4	4.2	4.2	5.2

**Table 2:** Deformation energy (kJ/mol) of the fine-grained nanocomposite.

	$\dot{\varepsilon }$/s^−1^	0.001	0.01	0.1	1
T/K					
523		107.80	110.20	158.02	177.30
573		107.31	109.71	157.31	176.50
623		86.49	88.42	126.79	142.26
673		69.26	70.81	101.54	113.92

**Table 3:** Hot deformation mechanism of the fine-grain nanocomposite.

	*n*	Q(kJ/mol)	Deformation Mechanisms
CN [13]	4.5	158	Dislocation climb resulting from lattice diffusion
FN	4.3	119	Dislocation climb resulting from grain boundary slip
